# DNA structural features and variability of complete MHC locus sequences

**DOI:** 10.3389/fbinf.2024.1392613

**Published:** 2024-07-03

**Authors:** Trudy M. Wassenaar, Terry Harville, Jonathan Chastain, Visanu Wanchai, David W. Ussery

**Affiliations:** ^1^ Molecular Microbiology and Genomics Consultants, Zotzenheim, Germany; ^2^ Department of Pathology and Laboratory Services, and Department of Internal Medicine, Division of Hematology, University of Arkansas for Medical Sciences, Little Rock, AR, United States; ^3^ Department of Pediatrics, The University of Arkansas for Medical Sciences University of Arkansas for Medical Sciences, Little Rock, AR, United States; ^4^ Myeloma Center, Winthrop P. Rockefeller Institute, Department of Internal Medicine, University of Arkansas for Medical Sciences, Little Rock, AR, United States; ^5^ Department of BioMedical Informatics, University of Arkansas for Medical Sciences, Little Rock, AR, United States

**Keywords:** MHC loci structural variability -revision 2.2MHC loci structural variability -revision 2.2 DNA structural atlas, MHC locus, HLA genes, visualization, polymorphisms

## Abstract

The major histocompatibility (MHC) locus, also known as the Human Leukocyte Antigen (HLA) genes, is located on the short arm of chromosome 6, and contains three regions (Class I, Class II and Class III). This 5 Mbp locus is one of the most variable regions of the human genome, yet it also encodes a set of highly conserved and important proteins related to immunological response. Genetic variations in this region are responsible for more diseases than in the entire rest of the human genome. However, information on local structural features of the DNA is largely ignored. With recent advances in long-read sequencing technology, it is now becoming possible to sequence the entire 5 Mbp MHC locus, producing complete diploid haplotypes of the whole region. Here, we describe structural maps based on the complete sequences from six different homozygous HLA cell lines. We find long-range structural variability in the different sequences for DNA stacking energy, position preference and curvature, variation in repeats, as well as more local changes in regions forming open chromatin structures, likely to influence gene expression levels. These structural maps can be useful in visualizing large scale structural variation across HLA types, in particular when this can be complemented with epigenetic signals.

## Introduction

The major histocompatibility (MHC) locus in humans is also known as the Human Leukocyte Antigen (HLA) locus, and is located on the short arm of chromosome 6. It is the most polymorphic region of the human genome, yet it also encodes a set of highly conserved and important proteins related to immunological response (Kulski et al., 2022; Medhasi and Chantratita, 2022; Turner et al., 2024). Genetic variations in this region are responsible for more diseases than in the entire rest of the human genome combined (Trowsdale and Knight, 2013; Medhasi and Chantratita, 2022). The total length of the MHC locus spans approximately 4.6 Mb–5 MB and contains over 200 genes. The MHC locus covers approximately the length of a typical bacterial genome, although a bacterial genome of that size would contain about 5,000 genes. In contrast, most of the human genome does not code for proteins, and the presence of 200 genes in 5 MB is considered “dense” by human genome standards.

Here, we first take a closer look at the highly polymorphic HLA genes and show how an atlas can visualize structural information of the local DNA, which can assist in interpretation of gene expression. We identify a need to add epigenetic signals as an extra layer of information, in order to truly capture the biological information stored in MHC DNA sequences.

### The polymorphic HLA genes located on the MHC locus

**FIGURE 1:**
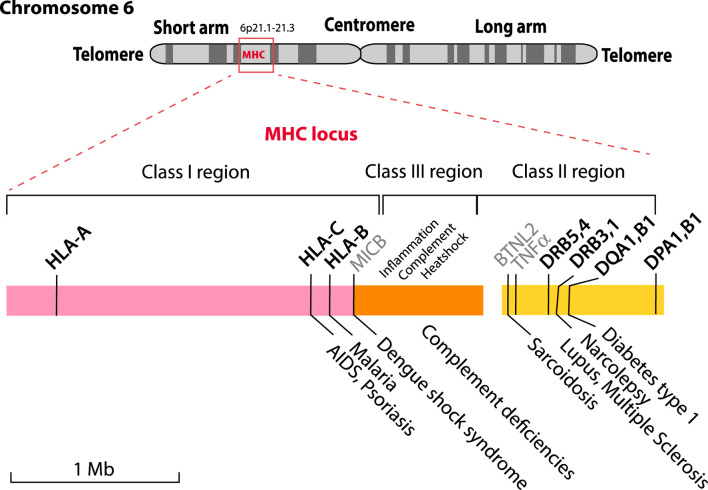
The MHC locus is located on the short arm of human Chromosome 6 with some of the HLA genes indicated. The locus is comprised of three genetic regions that encode components for Class I (pink), Class III (orange) and Class II (yellow) HLA proteins. The highly polymorphic proteins that are currently used for HLA typing are shown in bold above the colored blocks, together with MICB, BTNL2 and TNFα (shown in grey) that are not typically typed but are associated with diseases. The Class III region includes genes related to the keywords shown above it. A limited number of the known diseases associated with particular alleles are shown below the figure.

The MHC locus is divided into three regions called Class I, Class II and Class III. This historical nomenclature is based on the function of the HLA molecules these genes encode; now that this region has been sequenced, it turns out that Class III genes are located between Class I and Class II genes, as shown in Figure 1. Several recent review articles provide good starting point for background reading on HLA molecules (Kulski et al., 2022; Medhasi and Chantratita, 2022; Turner et al., 2024). Polymorphic Class I and Class II HLA molecules form protein complexes on the surface of cells that function in antigen presentation; these are regarded as the “classical” HLAs.

HLA Class I molecules are heterodimers consisting of one copy of a polymorphic heavy alpha chain (either HLA-A, HLA-B or HLA-C) that combine with one copy of the light chain beta-2 microglobulin [encoded by gene *B2M* which is located on chromosome 15 (Goodfellow et al., 1975)]. These complexes are present on essentially all cells (except for erythrocytes) and bind peptides that originate from degraded intracellular proteins; these are extracellularly presented to CD8^+^ T-cells, as part of the cellular immune response. This continuous antigen presentation process functions as a signal for cell viability and health, so that T-cells can remove infected or malfunctioning cells. The most strongly polymorphic component of the Class I HLA complex is the heavy chain, in particular the part of the protein that interacts with the peptide antigen; this region is encoded by exons two and three of the *HLA-A, HLA-B,* and *HLA-C* genes. Other parts of these proteins are strongly conserved, to ensure functionality. Multiple other genes and their encoded proteins contribute to a functional Class I system, and these may or may not be polymorphic.

HLA Class II complexes are also heterodimers consisting of an alpha protein and a beta protein (Lotteau et al., 1987). The alpha protein encoded by DQA1 combines with beta chain DQB1; alpha protein DPA1 combines with beta protein DPB1, and the beta proteins DRB1 to DRB5 combine with DRA, which is not polymorphic and is also encoded on chromosome 6. HLA Class II alpha chains contain much less variation compared to the beta chains. HLA Class II complexes are expressed on antigen-presenting immune cells such as dendritic cells, monocytes and B cells (Korman et al., 1985). Class II complexes are specialized to bind extra-cellular peptides, which arise from phagocytosis and intracellular processing for presentation on the cell surface to CD4^+^ helper T cells. Also located in the Class II region are genes coding proteins involved in the assembly of the HLA molecules.

The Class III region of the MHC locus contains genes that code for proteins not directly involved in antigen presentation, but more typically having a role in the immune response (Milner and Campbell, 2001). This region further includes genes for complement proteins, heatshock proteins and the tumor necrosis factor alpha (TNFα, gene name *TNF*), flanked by the gene *LTA* that codes for lymphotoxin α, formerly named TNF-β.

As Figure 1 illustrates for a small selection only, some alleles of MHC genes are associated with various diseases (Trowsdale and Knight, 2013; Medhasi and Chantratita, 2022). These include the clinical presentation of viral (HIV/AIDS, Dengue fever) and parasitic (malaria) infections, but also chronic conditions such as lupus, narcolepsy or type 1 diabetes, as well as autoimmune diseases (TNFα).

The HLA complexes are not only involved in defenses against infection, but also in organ rejection and the graft-versus-host disease response. This is in part due to too tight binding of the T cell’s T-cell Receptor (TcR) to a non-self HLA (any too tight binding to “self” HLA is removed by apoptosis during negative selection in the thymus, but this does not apply to non-self HLA). This makes HLA typing essential for compatible organ donor/recipient selection, and it is critical for hematopoietic cell transplantation. Classical serotyping for HLA determination has been replaced by molecular typing techniques. Commonly, reverse sequence specific oligonucleotide (rSSO) typing is performed, but, increasingly next-generation sequencing (NGS) is being utilized (Harville, 2009). Serologic typing was primarily limited to Class I, *HLA-A, HLA-B* and *HLA-C* and Class II, *HLA-DR*, and -DQ. Molecular typing includes all the components: *HLA-*A, -B, -C, DRB1, 3, *4, 5, DQA1, DQB1, DPA1,* and *DPB1*. These are marked in bold in Figure 1. The polymorphic nature of these genes is the result of single nucleotide polymorphisms (SNPs) and short insertions and deletions (indels) and in some cases alternative splicing or recombination. Exon two of the Class II genes is highly polymorphic, and can contain null-mutations, which result in truncated proteins, that might not be relevant for compatibility, and not be serologically detectable (Elsner and Blasczyk, 2004). This extensive genetic variation is the basis of the many alleles that have now been described and is collated in various databases, such as the Immuno Polymorphism Database (IPD) database (Barker et al., 2023), which currently lists more than 35,000 different MHC alleles.

It is only recently becoming possible to completely sequence both haplotypes, and to assess base modifications along the entire sequences (Wang et al., 2023). Regulation of the HLA protein levels on the surface of cells can involve many different mechanisms, as recently reviewed (Carey et al., 2019) including regulation by the trans-activators CIITA and NLRC5; promoter polymorphisms; activity of long-range promoters; variation in splicing; alternative start codons; alternate polyadenylation sites; DNA methylation; post-transcriptional regulation; physical stability and protein expression; and finally active internalization of HLA.

### Genome atlases can visualize DNA structural information

**FIGURE 2:**
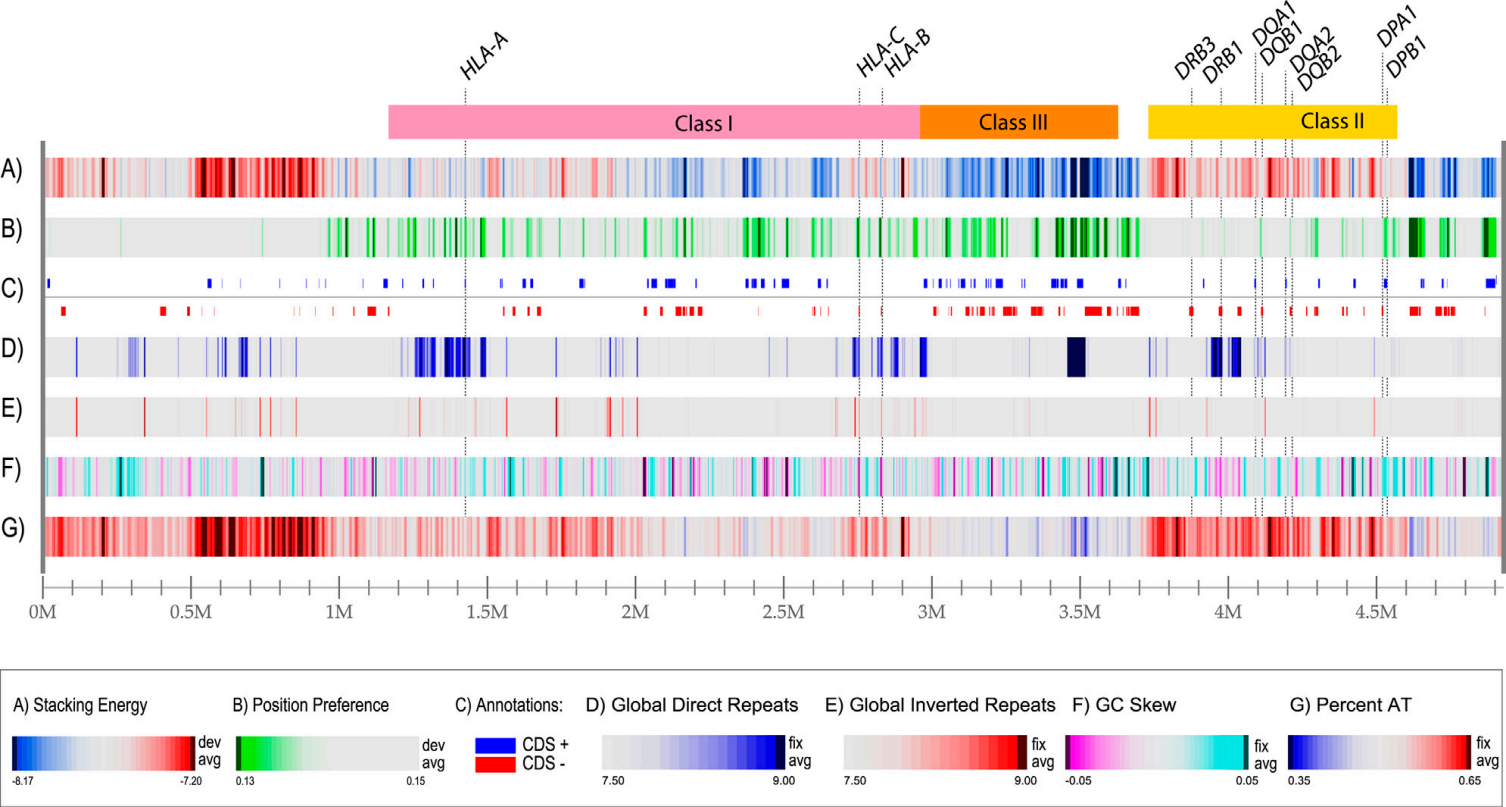
A structural atlas of the MHC locus of the MHC_APD cell line (GenBank accession number OK649231), a homozygous human cell line that is often used as a reference sequence. The MHC locus is represented as a linear atlas with six DNA structural lanes, and the three HLA regions of Figure 1 are indicated on top. Stacking energy is a measure how easily DNA will melt (dark red melts more easily). Position preference indicates the preference to wrap around nucleosomes (green indicates potentially open chromatin regions). Note, that all genes indicated lane C (separated for their location on the positive and negative strand, respectively) are shown with introns and exons combined. Genes for DRB2, DRB4 and DRB5 are absent in this cell line. Direct repeats are blue in lane D and Inverted repeats are red in land E. For this figure, the resolution is 4.9 Mbp divided by 2,500 (the width of the lane in pixels), which is 1972 bp/pixel.

In the past, we have developed tools to identify and visualize local DNA structures in bacterial genomes (Jensen et al., 1999; Pedersen et al., 2000; Ussery et al., 2001). These tools were used to produce a structural atlas for visualization of DNA structural information in the MHC locus, based on sequences derived from a homozygous cell line (Figure 2). The entire 5 Mbp region is represented in each of the lanes in the figure; the ‘resolution’ value is the number of bp represented by one pixel in the plot with a width of 2,500 pixels. Thus, a resolution value of 1972 bp means that the thinnest line in the plot represents almost 2000 bp. We use examples of homozygous cell lines here, instead of the human reference genome, as the latter originated from heterozygous cells that had been donated by multiple individuals (Lander et al., 2001). Lane A shows the local stacking energy, based on the amount of energy needed (in Kcal/mol) for a stretch of double-strand DNA to melt (Ornstein et al., 1978), whereby the smallest negative values, *i.e.*, red colors in lane A of Figure 2, represent DNA that melts more easily. Lane B shows nucleosomal position preference, which is a measure of anisotropic DNA flexibility (Satchwell et al., 1986), with lower values (green in lane B) indicating a decreased preference for wrapping around nucleosomes (Baldi et al., 1996; Willenbrock and Ussery, 2007). Lane C shows protein-coding genes in both orientations, here given as introns and exons combined. Lane D represents global direct repeats, calculated as the best match for a 100 bp window, scanned against the entire sequence; matches are binned into integer values, ranging from 9 (more than 90% identity) to 0 (less than 10% identity) and the values for each 100 bp window are plotted along the genome, starting at 50 bp, and ending 50 bp before the end of the genome (Jensen et al., 1999). Lane E shows global inverted repeats, calculated the same as before, except now the other strand of DNA is searched. Lane F gives the GC skew: a measure of the bias of G’s towards the sequenced strand, calculated as the running average of a 10,000 bp window, counting the number of G’s minus the number of C’s, divided by the total (Jensen et al., 1999). Lane G shows the AT-content, which is the fraction of A + T divided by the length of the window, which is usually set at 0.1% of the length plotted. These features are calculated using a sliding window, but the resolution of this atlas for the entire region is nearly 2000 nucleotides, so mutational events (SNPs, short indels) are not visible. Nevertheless, this combined graphical representation of structural properties makes several features obvious. For example, there is a large region upstream of the HLA Class I locus that will readily melt (bright red indicative of low stacking energy in lane A) and it is relatively AT-rich, as indicated by the red in lane G. This combination predicts it will have a higher mutational frequency. The region flanking the HLA Class II region at the other side of the locus has a low position preference (green stretches in lane B), combined with a high stacking energy (blue in lane A); this region is less likely to condense around nucleosomes, and forms a more open chromatin structure, so that genes located in this region have the potential to be highly expressed. The same applies to most of the HLA Class III region, that is also relatively low in AT content (high in GC content). The GC skew identifies some local regions producing a strong turquoise signal, as for DPA1/B1, which indicates a strong bias of G or C on the sequenced strand, or magenta, as for DRB1, meaning a strong bias of A and T on the sequenced strand; this is not related to the overall AT content of those regions.

### Genome atlases identify conservation in structural features between haplotypes

**FIGURE 3:**
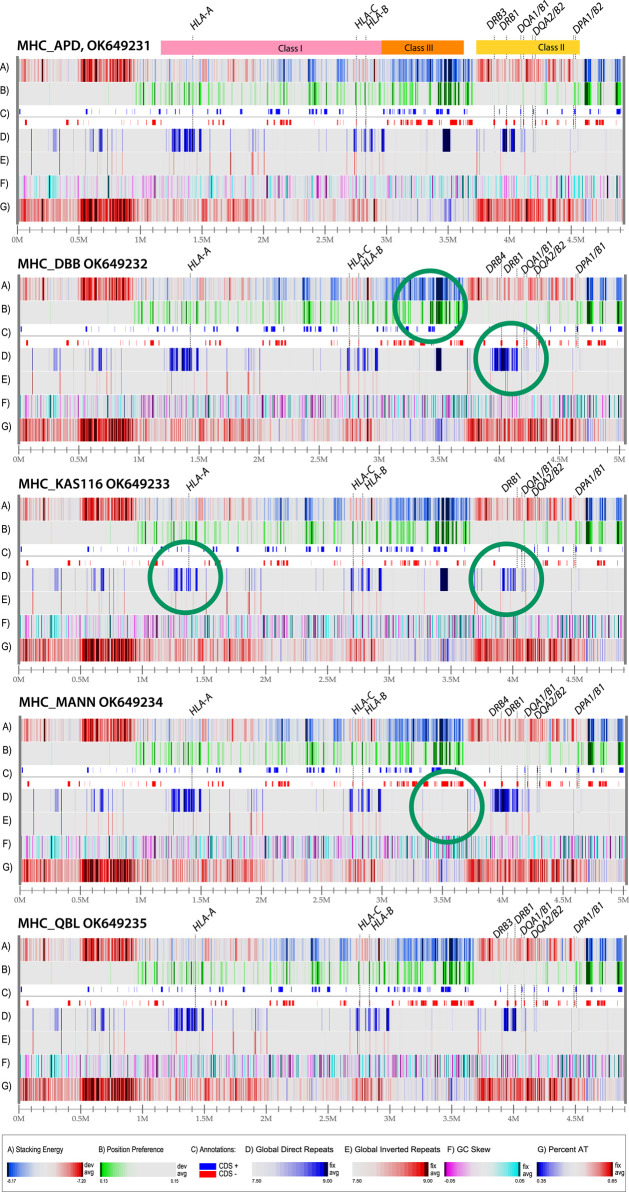
Structural atlases of the MHC locus of five homozygous cell lines. The three regions representing Class I, Class III and Class II genes are indicated at the top. The genes coding for proteins used for serological serotyping typing are indicated. Green circles indicate differences in structural features. The legends for the atlases are the same as in Figure 2 (dark red in lane A indicates regions that will melt; green in lane B indicates open chromatin regions, and direct repeats are blue in lane D, and inverted repeats red in lane E). The resolution is roughly the same for all five of the atlases, about 2 Kbp/pixel; the numbers varied from 1965 bp/pixel (MHC_KAS116) to 2020 bp/pixel (MHC_DBB).

The sequence that was used for construction of the atlas in Figure 2 was obtained from a homozygous cell line that has been made available for reference purposes (Houwaart et al., 2023). The MHC loci of other homozygous cell lines have been sequenced as well, and a comparison of five genome atlases from such cell lines is shown in Figure 3. The MHC Class I region contains a mixture of DNA melting profiles (lane A), and relatively few repeats (lands D and E). In contrast, the MHC Class III region is much more stable (blue in lane A) and contains a large direct repeat region (deep blue in lane D, around position 3.5 Mbp). In Figure 3, this repeat region is missing in the last two genomes. The MHC Class II region will melt more readily (red in lane A) and appears to have little open chromatin areas (lack of green in lane B). There are two direct repeats around 4 Mbp (dark blue in lane D) that is variable in the other genomes shown in Figure 3.

The five different genome atlases shown in Figure 3 are similar to the single genome atlas shown in Figure 2, with conservation of the structural features displayed at this level of resolution, including the strong AT-rich region with low stacking energy upstream of the HLA Class I region (dark red in lanes A and G, roughly between 0.5 and 1. Nevertheless, some variation is identified, such as in the direct repeat lane (lane D). Structural features that don’t coincide with genes may still be of relevance, for instance if they alter local DNA properties upstream of a gene. This may be the case for the observed variation in position preference upstream of *HLA-B* in the second genome in Figure 3, at position 2.9 Mbp, indicative of an open chromatin structure that supports high levels of transcription. Although this signal is approximately 10,000 bp removed from the gene start of *HLA-B*, this distance may still allow for cis-acting effects. For instance, a mutation positioned 13,910 bp upstream of the lactase gene present on Chromosome two is responsible for lactose tolerance in humans (Harris and Meyer, 2006). Some of the structural differences are highlighted with green circles in Figure 3. These represent obvious differences from the genomes above or below in the figure.

### Genome atlases identify structural features in promoter regions

**FIGURE 4:**
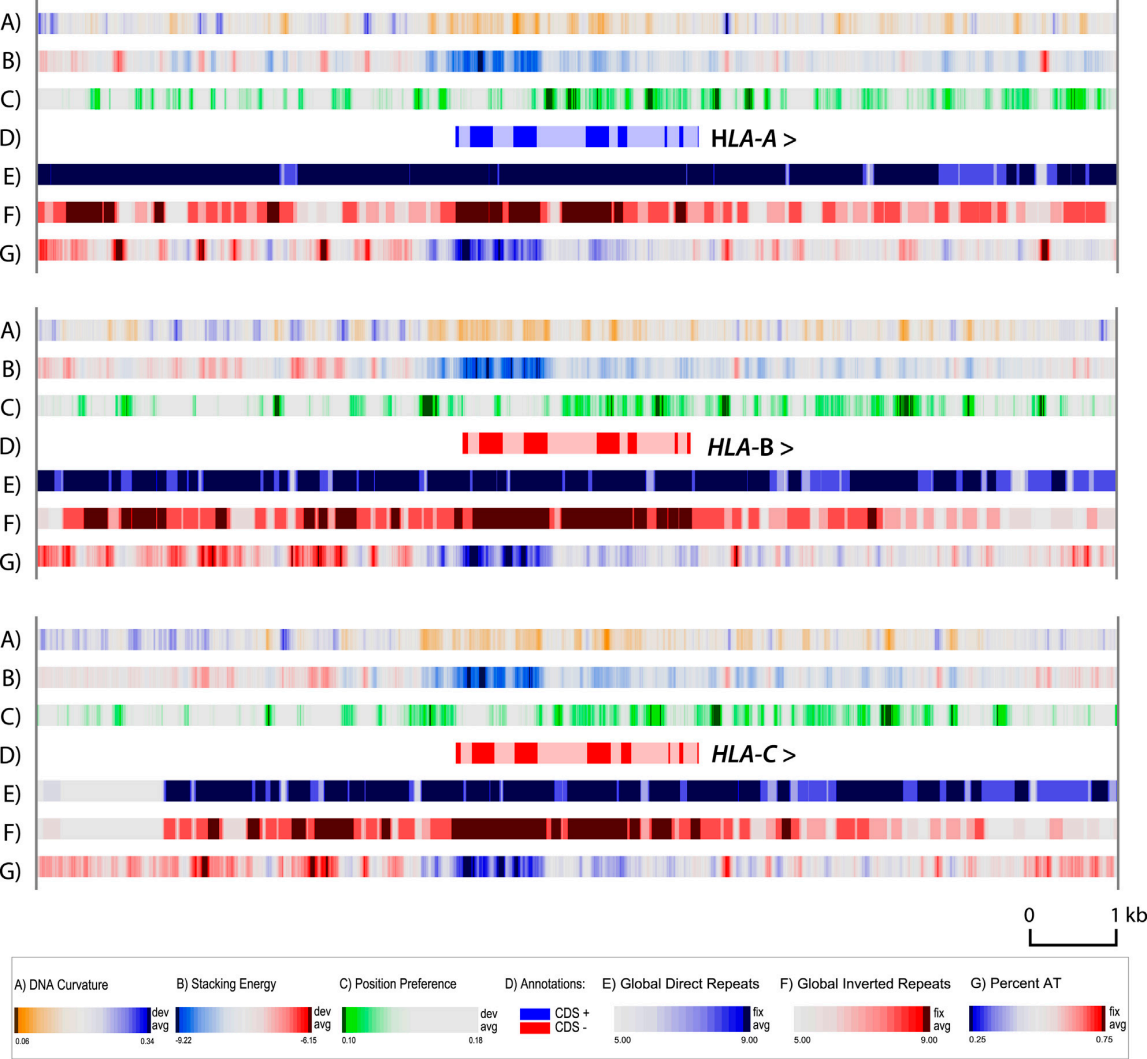
Zoom structural atlas of the *HLA-A*, *HLA-B* and *HLA-C* genes and surrounding sequences of MHC-APD. In this atlas, dark blue in lane A indicates regions with phased DNA curvature that can readily wrap around proteins; red in lane B indicates regions that will melt; green in lane C indicates open chromatin regions, and direct repeats are blue in lane E, and inverted repeats red in lane F. The genes are all shown in the 5′- to 3′- end orientation. Introns are shown in light shaded colors. Resolution: 6 bp.

A zoom atlas was produced with higher resolution for *HLA-A, HLA-B* and *HLA-C*. There was little variation between the cell lines for each of these genes (results not shown); but comparing these three genes with each other revealed some interesting features (Figure 4). This figure includes the same structural features as in Figures 2, 3, except we have removed the GC skew (lane F in the other figures) and added a lane for DNA curvature (lane A). The GC skew is a more global property, which can be used for determining the replication leading strand (since G’s are biased towards the leading strand), but provides little information in the zoom. DNA curvature is a more local structural characteristic, that can be helpful in locating promoter regions. Note that in general the upstream 5′ regions of all three *HLA* genes have curved regions (blue in lane A), which will help the DNA wrapping around the polymerase (Bansal et al., 2014), and also contain regions that will melt more readily (red in lane B). In the zoom in Figure 4, the position of introns and exons are indicated. The first third of all three genes has a higher GC-content (blue in lane G), matched by a stronger negative value in stacking energy (blue in lane B) which indicates that the DNA is more difficult to locally melt. This region is flanked by strong local position preference signals (green in lane C) with dark green regions within intron regions of all three of the HLA genes. Local strong position preference signals are also found downstream of all three *HLA* genes, although their distance to the stop codon varies. On this scale, with a resolution of six bp, the repeat lanes are heavily colored, as the global repeats already visible in Figure 3 are extending over much of the region shown in the zoom.

**FIGURE 5:**
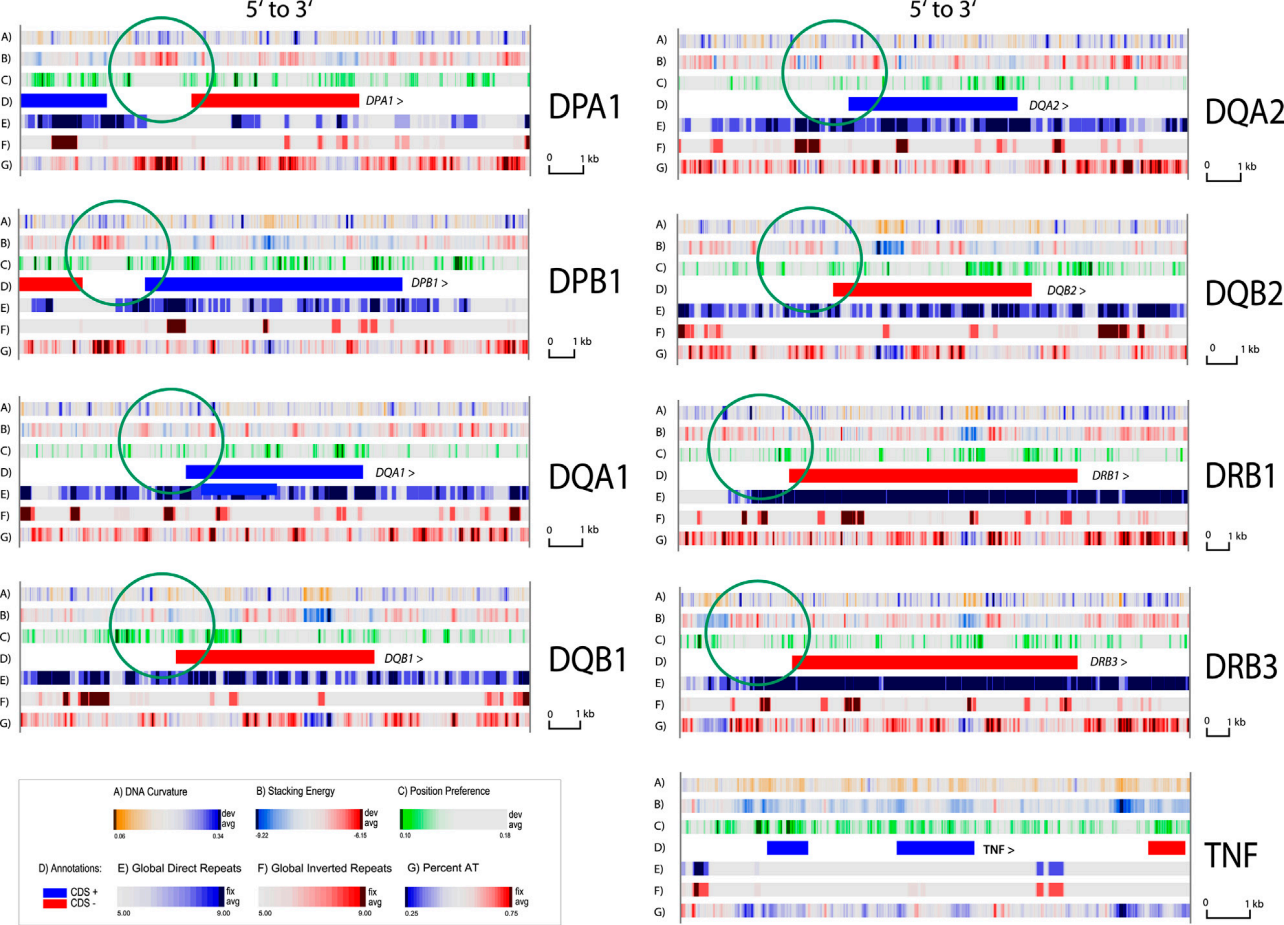
Zoom structural atlas of the other eight HLA genes. An atlas of the *TNF* gene is included for comparison. In this atlas, dark blue in lane A indicates regions with phased DNA curvature that can readily wrap around proteins; red in lane B indicates regions that will melt; green in lane C indicates open chromatin regions, and direct repeats are blue in lane E and inverted repeats red in lane F. The green circles indicate conserved structural features in the upstream region of the genes.

Zoom atlases of the other genes of interest were also produced (Figure 5). Here, green circles indicate conserved structural features directly upstream of the genes, typified by a localized less negative stacking energy (red in lane B) flanked by stronger negative values (blue). The curvature is also stronger here (blue in lane A). We hypothesize that these signals are related to the presence of promoter sequences. Promoters in eukaryotic DNA can be more difficult to predict than in bacterial DNA, and distal promoter elements can be distanced multiple kb away from the transcription start (Yella et al., 2018). However, the proximal promoter of eukaryotic genes is typically within 500 bp of the transcription start, and for divergently transcribed (as *DPA1* and *DPB1* in the figure) the promoter of both genes can be assumed to be present in the intergenic region. Indeed, the first two shown atlases of Figure 5 represent the same intergenic region, in two orientations, and we note the same pattern of structural signals in both. Interestingly, the gene *TNF*, which is located in the MHC class III region, does not contain this signal in its upstream region, and this might reflect a promoter further upstream.

## Future perspectives

With the advance of single molecule sequencing technology that produces long reads, it becomes possible to sequence large stretches of both chromosomal copies of the MHC loci. Genotyping of alleles is thus much more sensitive than serotyping to identify variation, but it may also produce noise. On the other hand, mutations in the promoter region of a gene may change its expression, and this can have consequences to the phenotype of that polymorphism. In such a case, the genetic signal may overlap with a serological signal that detects an antigen quantitatively.

Thus, allele descriptions provide more detailed information on a given gene than serology, but they fail to include two other levels of information that is also stored in DNA: epigenetic signals and structural features. Epigenetic signals are formed by (amongst others) modification of a DNA base, for instance the addition of a methyl group onto a cytosine base at a certain position. Gene expression within the MHC locus is strongly affected by epigenetics (Larsen and Alper, 2004). Single-molecule sequencing allows for reading of modified bases at the same time–for example, yielding six “letters” rather than four (G,A,T,C, and 5 mC and 5hmC; see George and Chinnaiyan, 2023). This adds an extra layer of information to that of DNA polymorphisms. Obviously, when a mutation (including silent mutations) involves a base that is normally methylated, the epigenetic information of the gene changes, and this may or may not change its expression. The picture may be even more complicated, as epigenetic signals can vary temporarily (depending on the developmental state of the cell and on external and environmental factors), as well as between different tissue types, and also between individuals. These base modification patterns can affect gene activity.

Another aspect to consider are local DNA structures, some of which are mapped in the structural atlases figures in this paper. In particular, open chromatin structures are known to be associated with highly expressed genes, and these are predicted as green areas in the “position preference” lane. Whereas the recognition of epigenetic signals that can be responsible for differences in gene activity is now more widely accepted, the effects of local DNA structural features are far less studied, although these can also severely affect gene activity. DNA structures are not readily visible from a DNA sequence, but properties like flexibility and melting can be calculated and predicted. This level of information, that is so far mostly often ignored in human genetics, deserves more attention.

Local DNA structures are dictated by their DNA sequence, so if a sequence changes, possibly the local DNA structure is changed, too. Changes in DNA structures can affect gene transcription, for instance by affecting the local condensation of the DNA. In addition, particular DNA structures, such as regions that melt more readily, making that location more vulnerable to mutations. We consider DNA structural features essential to fully describe the multi-level information stored in DNA sequences; we also hypothesize that highly polymorphic regions in a genome are likely associated with variation in DNA structural features. This hypothesis was put to the test here, with the MHC locus as a proof-of-principle.

## Conclusion

The use of structural atlases can assist in analysis of haplotype sequences of the MHC locus. We observe long-range structural variability in different haplotype sequences, as well as more local changes in regions forming open chromatin structures, likely to influence gene expression levels. These structural maps can be useful in visualizing large scale structural variation across HLA types. If this information can be combined with local epigenetic signals, a more complete picture of the biological information stored in DNA can be obtained, which we consider highly relevant to the complex and immunologically highly relevant MHC region.
